# Degradation Law of Mechanical Properties and Long-Term Compressive Strength Prediction Model of Unsaturated Polyester Resin Concrete in Aqueous Environments

**DOI:** 10.3390/polym18172112

**Published:** 2026-08-31

**Authors:** Wenchao Li, Fusheng Wen, Bin Han, Wenming Cao, Kai Liu

**Affiliations:** 1 College of Civil and Architectural Engineering, Taishan University, Taian 271000, China; 2 Shandong Engineering Research Center of High-Durability and Corrosion-Resistant New Building Materials, Taian 271000, China; 3 Changjiang Water Conservancy and Hydropower Engineering Construction (Wuhan) Co., Ltd., Jinan 250100, China

**Keywords:** unsaturated polyester resin concrete, aqueous environment, moisture diffusion, mechanical degradation, long-term strength prediction model

## Abstract

Unsaturated polyester resin concrete (UPC) exhibits high strength and corrosion resistance, and it has been widely used in hydraulic engineering structures. However, the diffusion of water molecules inevitably induces matrix plasticization and debonding at the aggregate–resin interface, which leads to progressive degradation of mechanical performance under long-term aqueous service conditions. To elucidate the water-induced mechanical deterioration mechanism of UPC and to develop a temperature-adaptive model for long-term compressive strength prediction, we prepared UPC specimens using graded quartz sand aggregate, an unsaturated polyester binder, V388 curing agent, and KH570 coupling agent at a fixed mass mixing ratio, followed by 7 days of natural curing after demolding. Accelerated water aging tests were conducted at three temperature levels (25 °C, 40 °C, 60 °C) and four immersion durations (15 d, 30 d, 45 d, 60 d), including water absorption, compressive, splitting tensile, and flexural tests. Based on Fick’s second diffusion law and the Arrhenius equation, we quantitatively analyzed moisture diffusion behavior and the evolution of mechanical degradation. The results indicate that the water absorption of UPC strictly follows Fickian diffusion, and that elevated temperature increases both the water absorption rate and the saturated water absorption capacity. The saturated water absorption ratios reached 0.15%, 0.16%, and 0.22% at 25 °C, 40 °C, and 60 °C, respectively, corresponding to apparent diffusion coefficients of 1.13 × 10^−6^, 1.67 × 10^−6^, and 4.52 × 10^−6^ mm/s. Long-term water aging progressively degrades the mechanical properties of UPC; after 60 d of immersion at 60 °C, the retention rates of compressive, splitting tensile, and flexural strength decreased to 88%, 85%, and 78%, respectively. We developed a physically coupled compressive strength prediction model based on moisture erosion depth and the associated reduction in effective bearing area. The ratio of model predictions to experimental data ranged from 0.93 to 0.98, indicating favorable conservative accuracy for engineering applications. When further combined with the Arrhenius relationship, the model enables extrapolation of the long-term mechanical properties of UPC under arbitrary service temperatures. This work provides theoretical support and a quantitative calculation framework for assessing the durability and predicting the service life of UPC hydraulic structures.

## 1. Introduction

Polymer concrete (PC) is composed of resin, aggregate, filler, curing agent, and coupling agent [1,2]. It exhibits characteristics such as high strength, good damping and shock absorption performance, corrosion resistance, and short curing time [3,4,5,6], and has been widely used in engineering fields such as buildings, roads and bridges, pavement repair, and pipelines [7,8,9,10]. Researchers have conducted experimental studies and theoretical analyses on the mechanical properties and durability of PC [11,12,13]. The resin content of PC is an important factor affecting its mechanical properties. For example, Yin et al. [14] showed that the compressive strength of PC decreases with the increase in effective resin content. Heidari-Rarani et al. [15] investigated the effects of aggregate shape, distribution, volume fraction, and interface parameters on the overall compressive performance of PC under uniaxial compression load. The excellent mechanical properties of PC mean that it can be widely applied in special environments. However, studies have shown that PC is not completely immune to all service environments, and its mechanical properties will degrade to varying degrees when exposed to acid, alkali, salt, freeze–thaw cycles, and damp heat [16,17,18]. Ghassemi et al. [19] studied the durability of epoxy polymer concrete, and the results showed that acidic environments cause the most damage to cement and polymer concrete. Heidarnezhad et al. [20] tested the mechanical properties of lightweight polymer concrete with different mix ratios at three temperatures (−15 °C, +5 °C, and +25 °C). The test results indicated that, as the temperature decreases from +25 °C to −15 °C, the compressive strength, splitting tensile strength, flexural strength, and elastic modulus of the material all increase, while energy absorption, impact energy, and ductility decrease.

There are many types of resin. Unsaturated polyester resin is cheap, and can be used as the cementitious material to make unsaturated polyester resin concrete (UPC) that is highly cost-effective and has wide applications [21]. In recent years, with the increasing application of UPC in water-related structures, research on its mechanical properties in aquatic environments has become particularly important [22,23,24]. The water absorbed by the UPC will affect the effective bonding of the aggregate–resin interface, degrade the resin, and thereby negatively impact the mechanical properties of UPC [25,26]. However, most previous investigations have focused on epoxy-based polymer concrete, while most studies on UPC report short-term experimental observations. A systematic understanding of its long-term mechanical degradation under aqueous conditions remains insufficient, and physically driven long-term strength prediction models are still scarce. UPC is widely used in hydraulic components, and its long-term mechanical performance directly determines structural service safety. The absence of reliable prediction methods means that durability evaluation is difficult. Based on this, this paper analyzes the degradation of compressive strength, tensile strength, and splitting tensile strength of UPC in water environments, and establishes a model for predicting compressive strength.

## 2. Test Materials and Methods

### 2.1. Test Materials

The aggregate used in the UPC was quartz sand, which was divided into four different groups according to particle size: 3–5 mm, 20–40 mesh, 40–70 mesh, and 70–120 mesh. Unsaturated polyester resin was selected as the cementitious material, V388 curing agent was used to accelerate the curing speed of UPC, and KH570 coupling agent was added to improve the interface bonding performance between aggregate and resin. The ratio of UPC aggregate to resin was determined according to the dosage in Table 1.

The specimen was prepared as follows:

(1) Weigh aggregates, resin, coupling agent, and curing agent according to the mix proportion;

(2) Add the coupling agent and curing agent to the resin and stir until uniform, and the resin changes from yellow to milky white;

(3) Add aggregates to the mixture and stir until uniform;

(4) Pour the UPC into the mold and compact it on a vibration table;

(5) Demold after standing for 3 days in a flat, dry and ventilated environment, and after natural curing for 7 days, treat according to the requirements of the UPC water absorption test and mechanical properties tests.

### 2.2. Test Method

(1) Water absorption test

**Figure 1:**
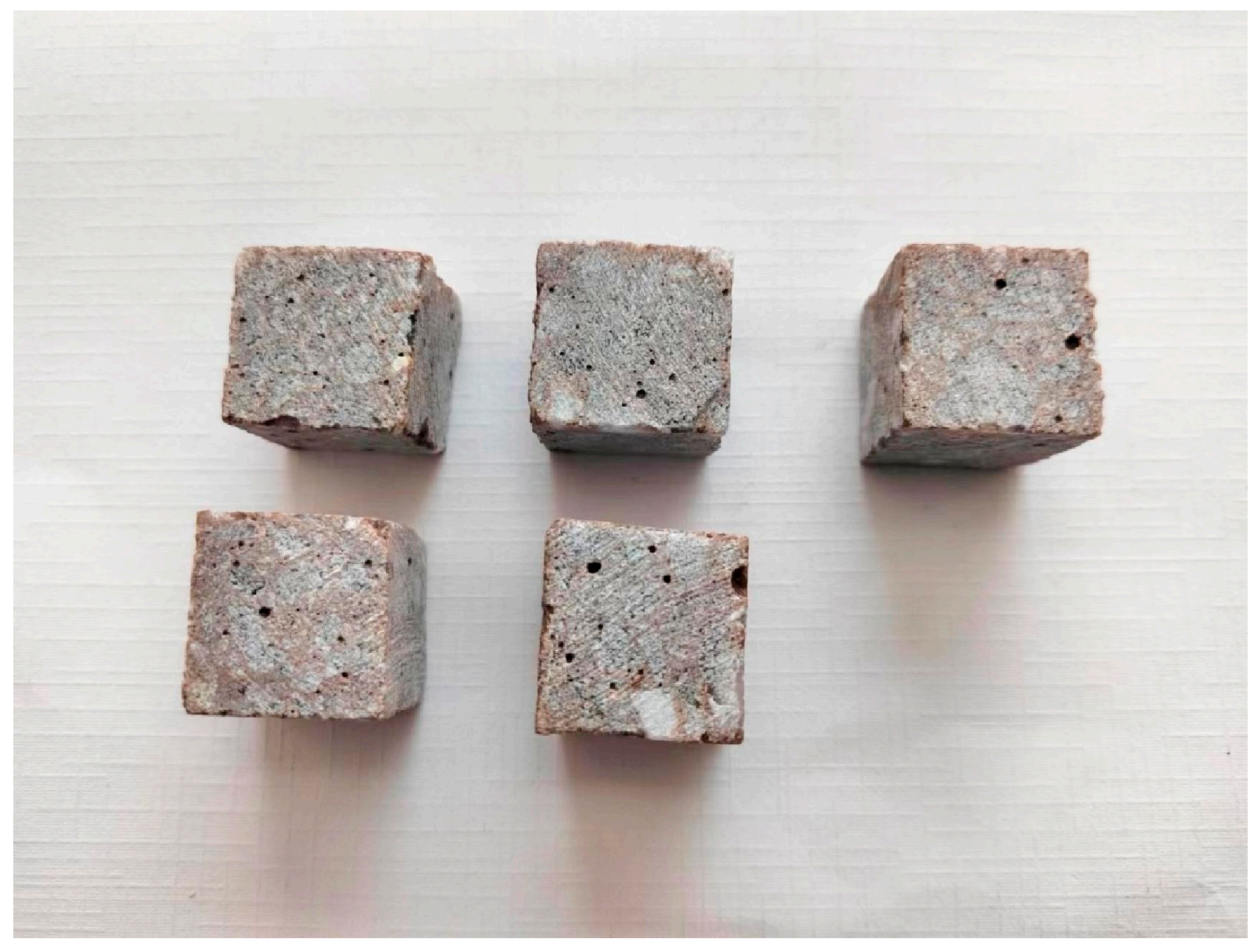
The UPC specimens used for the water absorption test.

The UPC was processed to a size of 10 mm × 10 mm × 10 mm, as shown in Figure 1, and dried in a drying oven to constant weight. Five samples were each soaked in water at 25 °C, 40 °C, and 60 °C. In the early stage of immersion (the first 30 days), the samples were taken out every day, and after wiping off the surface moisture, were weighed with a balance with an accuracy of 0.001 g. The water absorption rate was then calculated according to Equation (1). From 30 days to 60 days of immersion, they were tested once every three days.

(1) $$w=\frac{m_{s}-m_{0}}{m_{0}}\times 100\%$$

where *w* represents the water absorption rate of the sample, %; *m_s_* is the mass of the sample after water absorption, g; and *m*_0_ is the dry mass of the sample, g.

(2) Mechanical property test

The UPC specimens were immersed constant-temperature water bath tanks at three different aging temperatures: 25 °C, 40 °C, and 60 °C. When the aging period reached 15 d, 30 d, 45 d, and 60 d, the specimens were taken out for the compressive strength test, the splitting tensile strength test, and the flexural strength test, and the strength retention rate was tested [27].

**Figure 2:**
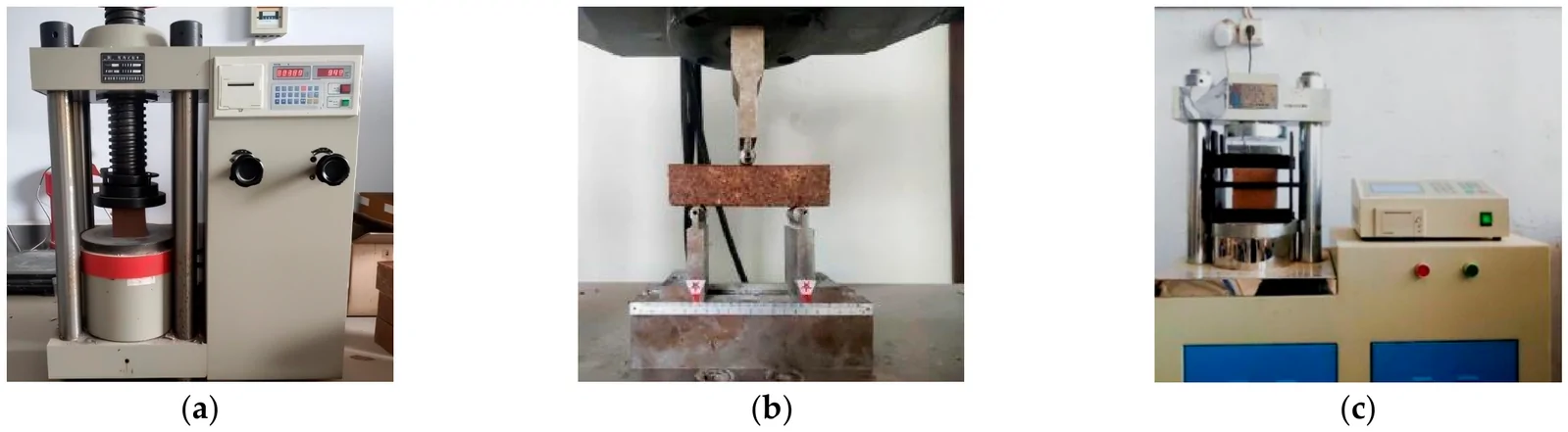
UPC mechanical properties test. (**a**) Compressive strength test; (**b**) flexural strength test; (**c**) split tensile strength test.

The mechanical property test is shown in Figure 2.

The splitting tensile strength test was conducted on a DYH-300B automatic pressure testing machine (Shandong Luda Test Instrument Co., Ltd., Tai’an, China), and the load at specimen failure was recorded. The splitting tensile strength was calculated according to Equation (2).

(2) $$f_{ts}=\frac{2F}{\pi A}=0.637\frac{F}{A}$$

where *f_ts_* is the splitting tensile strength of the cube specimen, MPa; *F* is the failure load of the specimen, N; and *A* is the splitting surface area of the specimen, mm^2^.

The flexural strength test was conducted using a WAW-1000D electro-hydraulic servo universal testing machine (Shandong Luda Test Instrument Co., Ltd., Tai’an, China)with a loading rate of 0.1 kN/s. The flexural strength was calculated according to Equation (3).

(3) $$R=\frac{3Fl}{2bh^{2}}$$

where *R* is the flexural strength of the specimen, MPa; *F* is the failure load of the specimen, N; *l* is the span between supports, mm; *b* is the cross-sectional width of the specimen, mm; and *h* is the cross-sectional height of the specimen, mm.

The compressive strength was tested using a YES-2000 pressure testing machine (Shandong Luda Test Instrument Co., Ltd., Tai’an, China) with a range of 2000 kN, and the compressive strength was calculated according to Equation (4).

(4) $$f_{cc}=\frac{F}{A}$$

where *f_cc_* is the compressive strength of cube specimens, MPa; *F* is the failure load of specimens, N; and *A* is the bearing area of specimens, mm^2^.

## 3. Test Results Analysis

**Figure 3:**
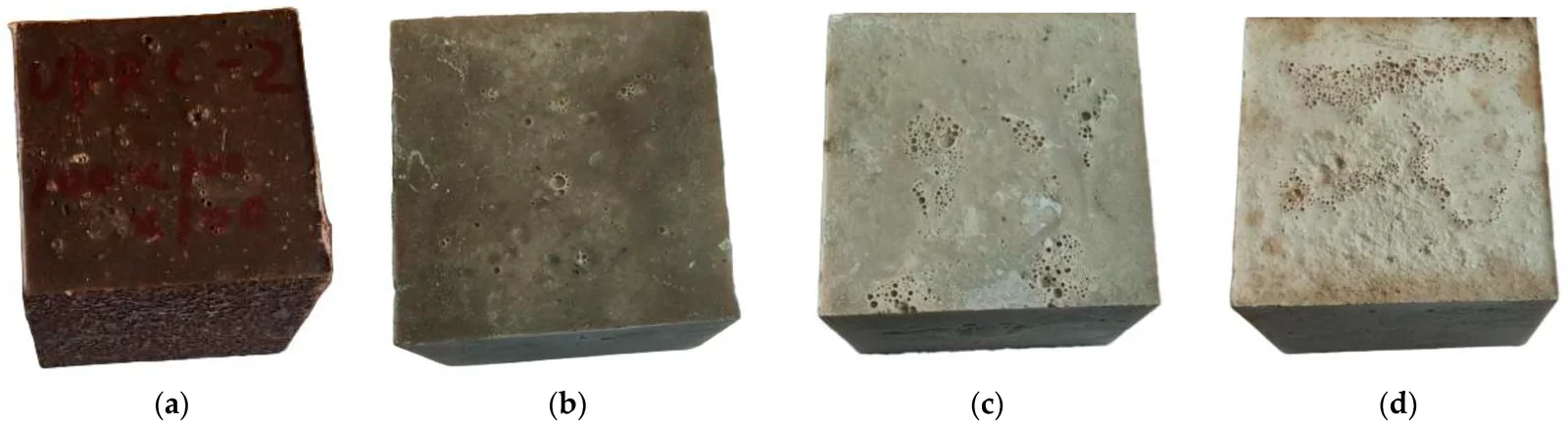
Appearance of specimens under different aging conditions: (**a**) Unaged specimen; (**b**) specimen aged at 25 °C for 60 d; (**c**) specimen aged at 40 °C for 60 d; (**d**) specimen aged at 60 °C for 60 d.

The surface of UPC specimens changed after immersion, with the most significant changes occurring among the specimens immersed at 60 °C for 60 days, with the surface color becoming lighter and traces of water stain intrusion to a certain depth. However, UPC specimens did not produce cracks, aggregate spalling or other damages under any aging conditions. The morphologies of specimens under different aging conditions are shown in Figure 3. This gradual surface lightening phenomenon is associated with both physical and chemical changes inside the material. Water molecules diffuse into the unsaturated polyester resin matrix and induce resin plasticization, accompanied by slight hydrolysis of ester groups at the resin–aggregate interface. From an engineering perspective, such color change can act as a qualitative auxiliary indicator for preliminarily assessing the water-induced deterioration of UPC components. Nevertheless, it cannot replace quantitative water absorption and mechanical property tests for complete durability evaluation.

### 3.1. Analysis of the Diffusion Test Results of Water in UPC

(1) Analysis of UPC water absorption test results

**Figure 4:**
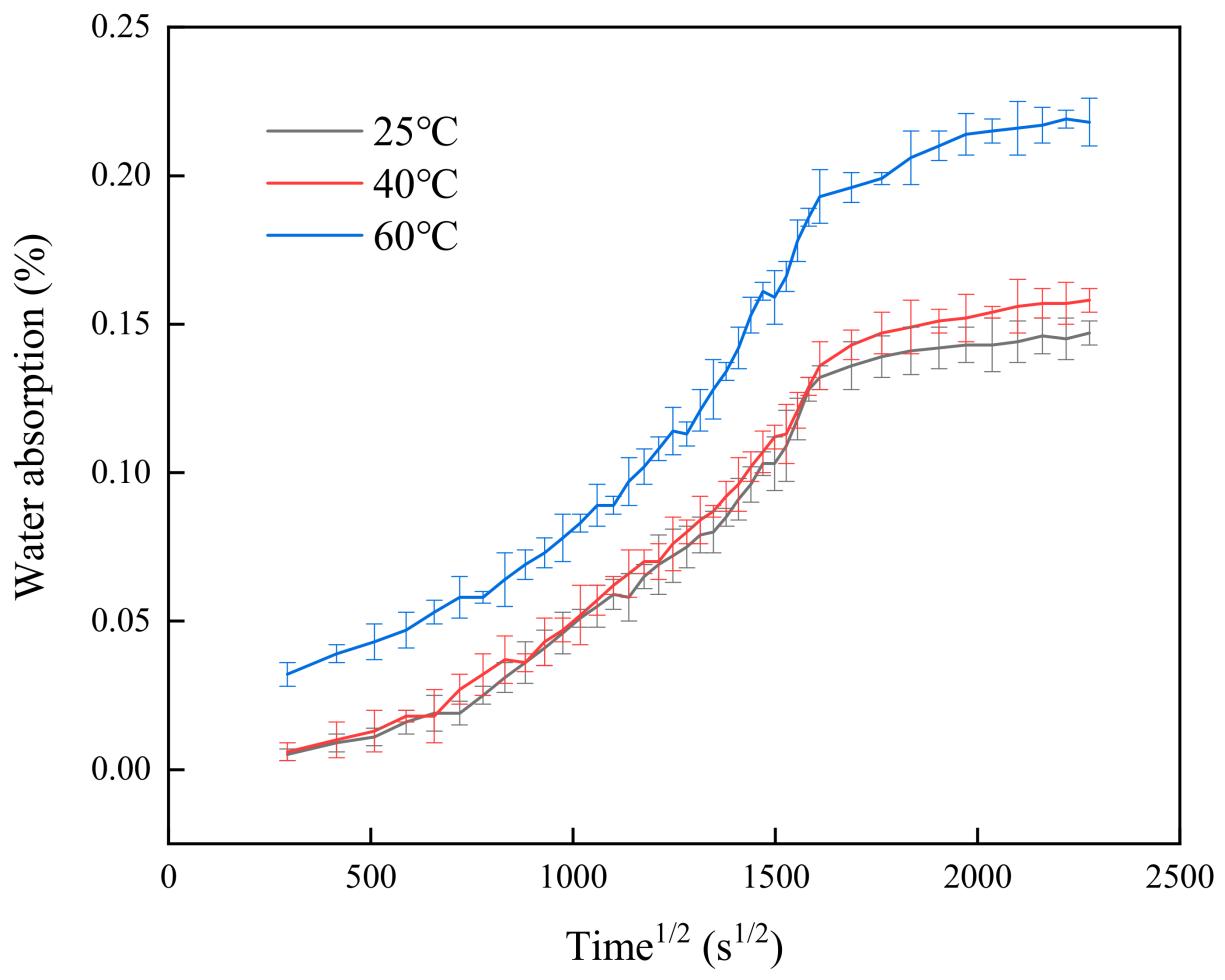
Water absorption curve of UPC under different aging conditions.

The water absorption rate of UPC was calculated according to Formula (1), and the test data was plotted in Figure 4. It can be seen from Figure 4 that the water absorption rate of UPC can be divided into two stages, which conforms to Fick’s diffusion law. In the early stage, the water absorption rate increases rapidly, and the water absorption rate has a linear relationship with the square root of the aging time. After aging for a certain period of time, the water absorption rate increases slowly, mainly because UPC tends to be in a saturated state, and the final water absorption rate remains around a certain value. At the same aging time, the water absorption rate of the UPC increased with the increase in temperature. The final water absorption rate also increased with the increase in temperature. When the temperature was 25 °C, 40 °C, and 60 °C, the final water content of the UPC was 0.15%,0.16% and 0.22%, respectively. The water absorption of UPC increases with immersion time, which can be attributed to water penetration and capillary action inside each component [28]. With increasing temperature, both the saturated water absorption and apparent diffusion coefficient of UPC rise. The thermal expansion of the resin matrix enlarges intermolecular voids, while high-temperature water exacerbates micro-debonding at the resin–aggregate interface and introduces additional micro-pores. At the same time, higher temperature improves the activity of water molecules and enhances diffusion transport [29].

(2) Calculation of apparent diffusion coefficient of UPC

The water absorption test piece used in this study was small, so the diffusion solution of Fick’s law in a finite-length object (Formula (5)) was selected [30]. Integrating Formula (5) over the size of the test piece in the diffusion direction, the equation of total water absorption can be obtained, as shown in Formula (6):

(5) $$C=C_{s}\{1-\frac{4}{\pi }\sum_{\nu =0}^{\infty }\frac{1}{2\nu +1}\cdot \sin[\frac{(2\nu +1)\pi }{h}\cdot x]\cdot \exp[-\frac{(2\nu +1{)}^{2}\pi^{2}}{h^{2}}Dt]\}$$

(6) $$\frac{M_{t}}{M_{s}}=1-\frac{8}{\pi^{2}}\sum_{\nu =0}^{\infty }\frac{1}{{\left(2\nu +1\right)}^{2}}\cdot \exp[-\frac{(2\nu +1{)}^{2}\pi^{2}}{h^{2}}Dt]$$

where M_t_ is the water absorption at any time; and M_s_ is the saturated water absorption of the specimen.

Previous research has shown that, in composites at the initial stage of immersion, Equation (6) can be simplified to Equation (7) [31]:

(7) $$\frac{M_{t}}{M_{s}}=\frac{2\sqrt{D}}{h\sqrt{\pi }}\sqrt{t}$$

It can be seen from the above equation that the water absorption at the initial stage of immersion has a linear relationship with the square root of time, which is consistent with the experimental results. By transforming Equation (7), the diffusion coefficient D during the diffusion of water molecules in the resin matrix composite can be obtained, as shown in Equation (8) [32,33]:

(8) $$D=\pi (\frac{h}{4M_{s}}{)}^{2}{\left(\frac{M_{t2}-M_{t1}}{\sqrt{t_{2}}-\sqrt{t_{1}}}\right)}^{2}$$

where M_t1_ and M_t2_ represent the water absorption corresponding to time $\sqrt{t_{2}}$ and $\sqrt{t_{1}}$, respectively.

Using Equation (8), the apparent diffusion coefficients in UPC at temperatures of 25 °C, 40 °C, and 60 °C are calculated to be 1.13 × 10^−6^ mm/s, 1.67 × 10^−6^ mm/s, and 4.52 × 10^−6^ mm/s, respectively.

(3) Calculation of UPC moisture diffusion depth

Moisture diffusion depth has a significant impact on the strength of UPC specimens. During the soaking process of mechanical performance specimens, a certain depth below the specimen surface is significantly affected. Therefore, the diffusion solution of semi-infinite objects based on Fick’s law is used to analyze the influence of UPC in water environment. At any given time t, the erosion depth of UPC can be expressed as follows:

(9) $$x=2\sqrt{Dt}\cdot {\mathrm{erf}}^{-1}(1-\frac{w}{w_{s}})$$

where x is the erosion depth, mm; w is the water absorption at position x at time t; and ws is the water absorption at the surface of the specimen.

### 3.2. Analysis of Test Results on Mechanical Properties of UPC in Water Environment

**Figure 5:**
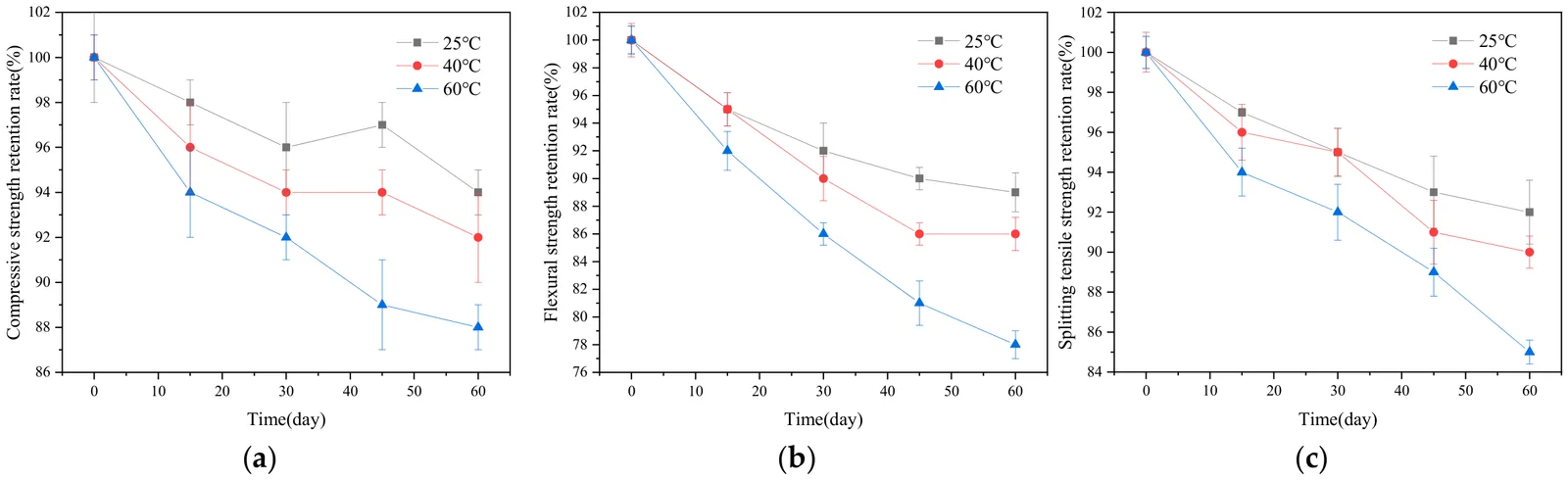
The mechanical properties of UPC under different aging conditions vary with aging time. (**a**) Compressive strength; (**b**) flexural strength; (**c**) splitting tensile strength.

The mechanical properties of UPC specimens without aging and with aging periods of 15 d, 30 d, 45 d and 60 d were compared. The compressive strength, flexural strength, and splitting tensile strength of unaged UPC specimens were 119.37 MPa, 22.66 MPa, and 7.65 MPa, respectively. The strength retention rates of UPC after different aging conditions are shown in Figure 5.

A summary and analysis of the variation in UPC performance in an aquatic environment is given below and in Figure 5:

(1) In water, the mechanical properties of UPC decline with the increase in temperature (except for the flexural strength after aging at 25 and 40 °C for 15 d and the splitting tensile strength after aging at 25 and 40 °C for 30 d). For example, after exposure of UPC aged for 60 d to 25 °C, 40 °C, and 60 °C, its flexural strength retention rates were 89%, 86%, and 78%, respectively, the compressive strength retention rates were 94%, 92%, and 88%, and the splitting tensile strength retention rates were 92%, 90%, and 85%. The main reason for this is that the increase in temperature can accelerate the diffusion of water molecules in UPC and speed up the erosion of the resin matrix and the interface bonding between aggregate and resin matrix, thereby accelerating the reduction in mechanical properties.

(2) The mechanical properties of UPC gradually decrease with the increase in aging time (except for the change in compressive strength of UPC from 30 d to 45 d when aged at 25 and 40 °C). As aging time increased, the cumulative amount of water in UPC increased, resulting in a greater decrease in UPC performance. At the same time, the test results show that the rate of decline was faster in the early stage of aging. Taking the flexural strength of UPC at 60 °C as an example, the strength decreased by 14% from 0 to 30 d, and by 8% from 30 to 60 d. The main reason for this phenomenon is that UPC absorbs water quickly in the early stage of aging, and the water absorption rate decreases significantly after approaching saturation.

(3) Under the same conditions, the retention rate of flexural strength is significantly lower than that of compressive strength and splitting tensile strength. After aging at 60 °C for 60 d, the flexural strength retention rate of UPC is 78%, which is lower than the compressive strength retention rate (88%) and splitting tensile strength retention rate (85%). The main reason is that the cross-sectional area of the specimen used for testing flexural strength is smaller than that of the other two tests, and so the erosion by water molecules will have a more significant impact on the results. The effect of water aging on splitting tensile strength is slightly great than that on compressive strength, which can be explained by the fact that aging increases the brittleness of UPC, making it more susceptible to minor damage.

This study demonstrates that the mechanical properties of UPC degrade gradually with increasing immersion duration and temperature, which is consistent with the findings reported in the previous literature [34,35,36]. Water molecules diffuse into the internal porous structure of UPC, plasticize the unsaturated polyester resin matrix, and weaken the intermolecular interactions within the resin. Concurrently, moisture preferentially penetrates the interfacial transition zone between the resin matrix and aggregate, triggering interfacial micro-delamination and generating micropore defects. The synergistic effect of hygrothermal exposure further amplifies the additional internal stresses induced by the thermal expansion mismatch between the resin and aggregate, which accelerates the propagation of microcracks. Consequently, tensile properties including splitting tensile strength and flexural strength—both of which are highly dependent on interfacial bonding conditions—exhibit significantly more pronounced strength degradation than compressive strength [37,38].

## 4. Long-Term Mechanical Performance Model of UPC

Most previous research on the durability of UPC in aquatic environments relies on empirical fitting of strength–time and strength–moisture relationships under fixed temperatures, whereas physically based predictive models remain very limited [35,39]. In contrast, for polymer-matrix composites—including fiber-reinforced polymers (FRP)—moisture diffusion is routinely modeled using Fick’s second law, while temperature-dependent degradation kinetics are quantified via the Arrhenius equation [27,40,41,42]. Although these two physical theories are widely available, they are seldom jointly applied to describe UPC degradation. Existing studies merely perform empirical fitting on experimental data and cannot quantitatively describe how the expanding moisture-damaged zone reduces the effective load-bearing cross-section. Building upon these well-established physical frameworks, this study proposes a physics-coupled model to predict long-term compressive strength loss in UPC. Different from pure empirical formulas, this model explicitly links moisture penetration depth governed by Fickian diffusion to thermally activated degradation via Arrhenius correction. Its key improvement is that the advancing moisture-affected zone is further quantitatively correlated with the progressive reduction in effective load-bearing cross-section of UPC, so as to realize long-term strength prediction under variable temperature immersion conditions.

To establish the long-term mechanical performance model of the compressive strength of UPC in water, it is assumed that the strength within the range significantly eroded by water molecules is zero, and the strength of the un-eroded part remains unchanged [43]. The erosion depth x is determined by Equation (9). When calculating the erosion depth x, it is assumed that significant water diffusion occurs when the water absorption w reaches half of the surface water absorption w_s_, and the UPC strength in the area from here to the specimen surface is zero, while the strength in other areas remains unchanged. The erosion depth x of the significant diffusion area (w = 0.5w_s_) can be obtained from Equation (10):

(10) $$x=\sqrt{Dt}$$

**Figure 6:**
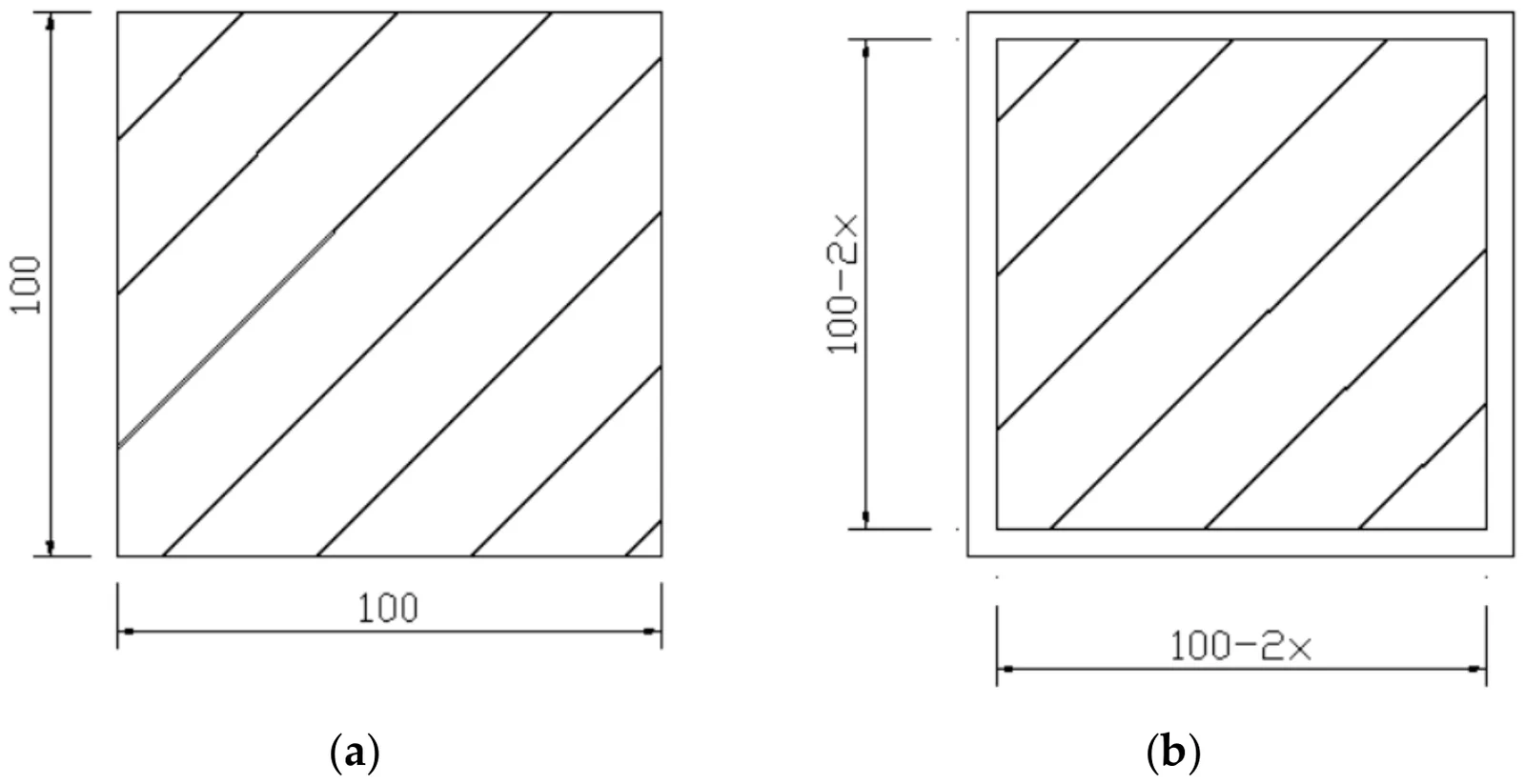
Comparison of effective force cross sections of UPC before and after erosion. (**a**) Before aging; (**b**) after aging.

The comparison of the stressed cross-sectional areas of the specimens before and after erosion is shown in Figure 6.

Now, the calculation formula for the compressive strength of UPC after erosion is determined according to the erosion depth. Assuming that the compressive strengths of UPC before and after erosion are f_0_ and f_x,_ respectively, two strength relationship expressions are obtained as follows, according to Formula (4):

(11) $$f_{x}=f_{0}\times (h-2\sqrt{Dt}{)}^{2}/h^{2}$$

**Figure 7:**
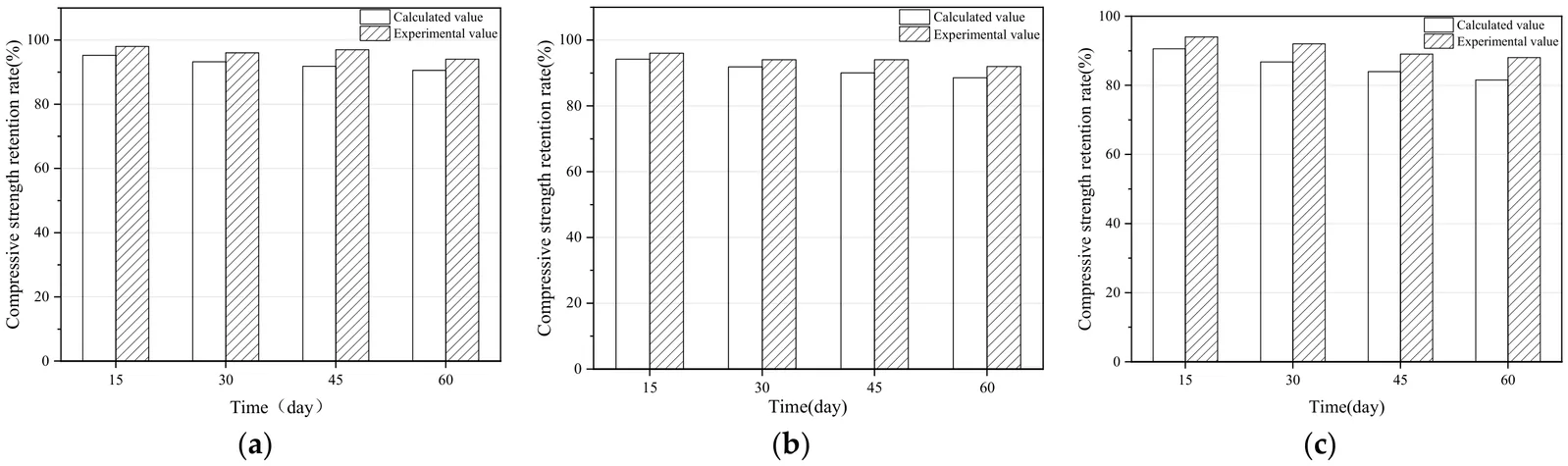
Comparison of the calculated and experimental values of UPC compressive strength retention under different aging conditions. (**a**) 25 °C; (**b**) 40 °C; (**c**) 60 °C.

The data obtained from the experiment are compared with the calculated data from Formula (11), as shown in Figure 7.

It can be seen from Figure 7 that the calculated value of the UPC compressive strength retention rate is slightly smaller than the experimental value. The reason for this phenomenon may be that the UPC still retains a certain strength within the range obviously eroded by water molecules, while the model assumes that the UPC strength in this area is zero, so the calculated results of the model are smaller. However, considering that the range of obvious erosion is much smaller than the size of the UPC compressive specimen and the model calculation results are conservative, it is acceptable in engineering. After calculation, the ratio of the calculated value to the experimental value ranges from 0.93 to 0.98, indicating that the method described in this study is highly accurate at predicting the compressive strength of UPC in water. In this study, the mechanical properties of UPC at 3 different temperatures were analyzed. Considering the diversity of UPC service temperatures in actual engineering, we analyzed the performance of UPC in water environments at all different temperatures. It can be seen from Equation (11) that the mechanical property degradation of UPC in a water environment mainly depends on the diffusion coefficient and aging time. Among them, the diffusion coefficient D largely determines the diffusion rate of water molecules in UPC. The diffusion coefficient D is mainly related to the aging temperature, and its relationship can be expressed by the Arrhenius equation [31], as shown in Formula (12).

(12) $$D=D_{0}\exp(-Q/RT)$$

where *D* is the diffusion constant, which depends on the material composition and structure and is independent of temperature; *Q* is the diffusion activation energy, which is independent of temperature, J/mol; *R* is the gas constant, J/mol·K; and *T* is the temperature, K.

When the temperature is constant, D_0_ and Q can be regarded as constants. Taking the natural logarithm of both sides of Formula (12), the following formula can be obtained:

(13) $$\ln D=\ln D_{0}-\frac{Q}{R}\cdot \frac{1}{T}$$

**Figure 8:**
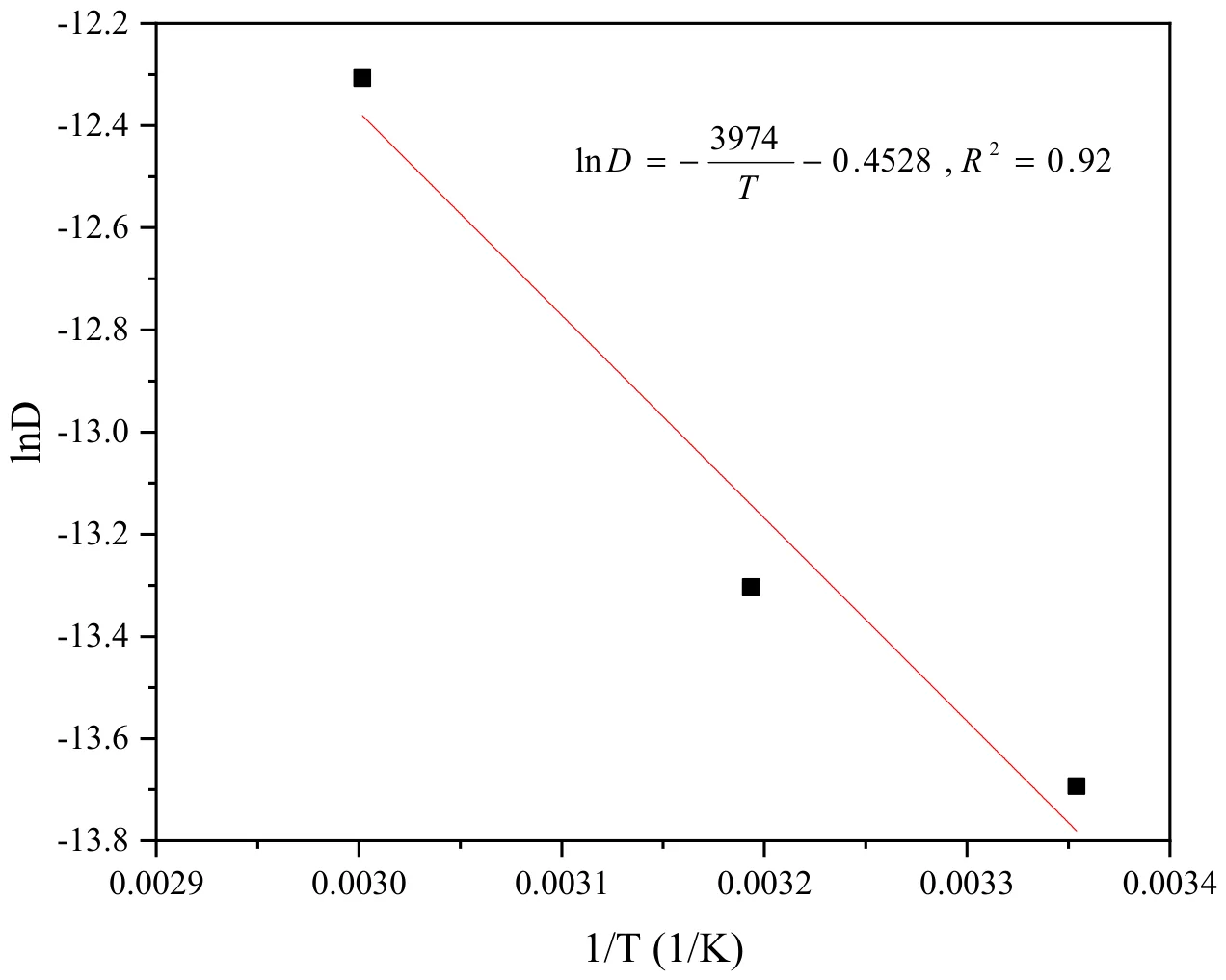
The relationship between the diffusion coefficient and temperature.

The natural logarithm of the diffusion coefficient has a linear relationship with the reciprocal of temperature. Therefore, the diffusion coefficient at any temperature can be calculated by measuring the diffusion coefficients at two or more temperatures. The diffusion coefficients at temperatures of 25, 40, and 60 °C were obtained experimentally, and Formula (13) was fitted, as shown in Figure 8.

By substituting the temperature of the location where UPC is in service into the fitting formula in Figure 8, the diffusion coefficient at any temperature can be calculated. To predict the compressive strength of UPC when serving in an aquatic environment, the following procedure can be followed:

(1) Use Fick’s second law to determine the region where UPC is significantly affected by water molecule diffusion, which can be calculated according to Formula (9). When the set water content reaches half of the final water content or the surface water content as the significant diffusion region, it can be calculated according to Formula (10);

(2) Obtain the relational expression between the diffusion coefficient and temperature according to the Arrhenius equation. Based on the experimentally measured diffusion coefficients of water molecules in UPC (at least two data points), fit the constants in Formula (13);

(3) Determine the temperature of the area where UPC is in service, substitute it into the fitting formula obtained in step (2) to find the diffusion coefficient at that temperature, and then substitute it into Formula (11) to obtain the compressive strength degradation curve of UPC when serving in the aqueous environment of that area.

The proposed prediction model has specific limitations in its applicability and validation scope. It is valid for unsaturated polyester resin concrete with the tested mix proportion under pure water immersion conditions. An idealized assumption in the model is that the moisture-damaged zone is treated as a zero-strength region; in practice, however, the material exhibits gradual degradation in stiffness and strength rather than complete failure. Therefore, this model should be applied with caution to other mix proportions or to exposure in corrosive solutions.

## 5. Conclusions

This paper reports an experimental study on the degradation of the mechanical properties of UPC in an aquatic environment. The main results are summarized as follows:

(1) The water absorption performance of UPC in an aquatic environment is related to temperature. As the temperature increases, both the water absorption rate and saturated water absorption of UPC increase. When the temperatures were 25 °C, 40 °C, and 60 °C, the final water contents of the UPC were 0.15%, 0.16% and 0.22%, respectively. The diffusion of water molecules in UPC conforms to Fick’s second law. In the early stage of aging, the water absorption rate increases rapidly, and it has a linear relationship with the square root of aging time. After aging for a certain period of time, the water absorption rate increases slowly and finally tends to a fixed value.

(2) The calculation formula for the diffusion coefficient of water molecules in UPC was obtained using Fick’s second law. The diffusion coefficient is significantly affected by temperature. The calculated apparent diffusion coefficients of water molecules in the UPC at temperatures of 25 °C, 40 °C, and 60 °C were 1.13 × 10^−6^ mm/s, 1.67 × 10^−6^ mm/s, and 4.52 × 10^−6^ mm/s, respectively. In addition, the formula for calculating the erosion depth of UPC affected by water molecules was obtained, which is related to the aging time and diffusion coefficient.

(3) The compressive strength, flexural strength, and splitting tensile strength of the UPC in the aquatic environment at different temperatures were analyzed. The increase in temperature can accelerate the diffusion of water molecules in UPC and accelerate the erosion of the UPC resin matrix and the interface bonding between aggregate and resin matrix, thereby causing an increase in the degradation rate of mechanical properties. UPC absorbs water quickly in the early stage of aging, and the water absorption rate decreases significantly after approaching the saturated state, making the performance degrade faster in the early stage of aging than in the later stage. Under the same conditions, the retention rate of flexural strength is significantly lower than that of compressive strength and splitting tensile strength.

(4) A model for predicting the mechanical properties of UPC during service in an aquatic environment was established based on Fick’s second law and the Arrhenius equation. The calculated value of the model is slightly smaller than the experimental value, and the ratio of the compressive strength value calculated by the model to the experimental value ranges from 0.93 to 0.98, indicating that the model has high reliability and that the calculation results are conservative and can be used in engineering. The possible reason for this phenomenon is that UPC still retains a certain strength in the range significantly eroded by water molecules, but the model assumes that this part of the strength is zero during calculation.

(5) A procedure for establishing the mechanical property degradation model of UPC when serving in any region was determined. First, calculate the thickness of the significantly eroded area according to Formula (9) or (10); then, obtain the diffusion coefficients of at least two temperatures through experiments, use the Arrhenius equation to find the diffusion coefficient at the temperature of the service area according to Formula (13), and substitute it into the mechanical property formula to calculate the change in mechanical properties with time.

## Figures and Tables

**Table 1:** Mixing ratios of the UPC specimen.

Resin	3–5 mm Quartz Sand	20–40 Mesh Quartz Sand	40–70 Mesh Quartz Sand	70–120 Mesh Quartz Sand	Coupling Agent	Curing Agent
1	2.83	1.89	0.94	0.63	1%	1.5%

## Data Availability

Data is contained within the article.

## Abbreviations

The following abbreviations are used in this manuscript:

UPC	Polymer Unsaturated Polyester resin Concrete
PC	Polymer concrete
